# MXene-Based Photocatalysts in Degradation of Organic and Pharmaceutical Pollutants

**DOI:** 10.3390/molecules27206939

**Published:** 2022-10-16

**Authors:** Siavash Iravani, Rajender S. Varma

**Affiliations:** 1 Faculty of Pharmacy and Pharmaceutical Sciences, Isfahan University of Medical Sciences, Isfahan 81746-73461, Iran; 2 Regional Centre of Advanced Technologies and Materials, Czech Advanced Technology and Research Institute, Palacký University in Olomouc, Šlechtitelů 27, 783 71 Olomouc, Czech Republic

**Keywords:** photocatalysis, MXenes, pharmaceutical pollutants, photocatalytic degradation, pollutants, MXene-based nanocomposites

## Abstract

These days, explorations have focused on designing two-dimensional (2D) nanomaterials with useful (photo)catalytic and environmental applications. Among them, MXene-based composites have garnered great attention owing to their unique optical, mechanical, thermal, chemical, and electronic properties. Various MXene-based photocatalysts have been inventively constructed for a variety of photocatalytic applications ranging from pollutant degradation to hydrogen evolution. They can be applied as co-catalysts in combination with assorted common photocatalysts such as metal sulfide, metal oxides, metal–organic frameworks, graphene, and graphitic carbon nitride to enhance the function of photocatalytic removal of organic/pharmaceutical pollutants, nitrogen fixation, photocatalytic hydrogen evolution, and carbon dioxide conversion, among others. High electrical conductivity, robust photothermal effects, large surface area, hydrophilicity, and abundant surface functional groups of MXenes render them as attractive candidates for photocatalytic removal of pollutants as well as improvement of photocatalytic performance of semiconductor catalysts. Herein, the most recent developments in photocatalytic degradation of organic and pharmaceutical pollutants using MXene-based composites are deliberated, with a focus on important challenges and future perspectives; techniques for fabrication of these photocatalysts are also covered.

## 1. Introduction

**Figure 1:**
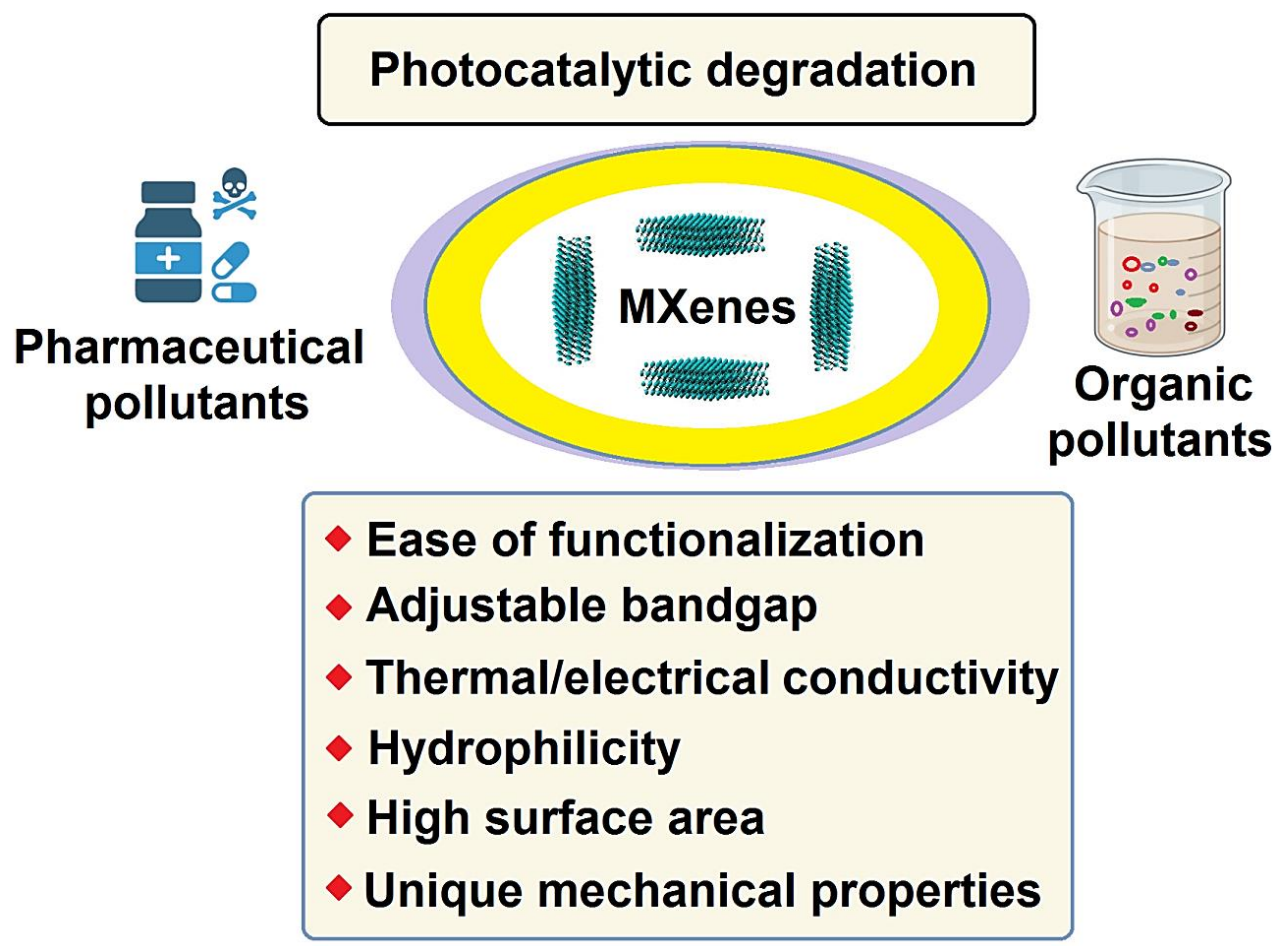
MXenes with unique properties for photocatalytic degradation of pharmaceuticals and organic pollutants.

Two-dimensional (2D) nanomaterials have been broadly explored by researchers for their unique catalytic and biomedical applications [1,2,3,4,5]. Among them, MXene-based composites have received immense attention owing to their attractive physicochemical features [6,7,8]. MXenes (with a typical formula of M_n+1_X_n_T_x_, n = 1–3) have been widely synthesized using a variety of bottom–up and top–down strategies [9,10,11,12], including acid etching, chemical vapor deposition, electrochemical etching, molten salts etching, hydrothermal method, solvothermal techniques, ball-Milling, ultrasonic synthesis, pyrolysis approaches, among others [13,14,15,16,17]. However, several aspects pertaining to the application of non-/less-toxic agents, the optimal environmentally benign conditions, the improvement of stability/structural defects, the oxidation of MXenes, delamination methods, and the elimination of Al etching by-products ought to be further explored [18,19,20,21,22,23]. MXene-based structures with a large surface area, surface controllable chemical characteristics, regular planar structures, unique optical/thermal features, hydrophilicity, excellent metal conductivity, and abundant derivatives have been widely studied for photocatalytic degradation of pollutants (Figure 1) [24,25,26,27,28]. They have shown excellent potential for eliminating pollutants through interfacial chemical transformation and sorption, along with catalytic removal and photocatalytic degradation capabilities [29,30].

MXenes with high conductivity and unique lamellar nanostructures can enhance the photo-electrocatalytic capabilities of composites as co-catalysts [31,32]. Among MXenes, titanium carbide (Ti_3_C_2_T_x_) with promising photocatalytic performance, flexible structure, electronic features, and semimetal nature has been broadly studied for environmental applications, especially photocatalytic degradation of hazardous pollutants (Table 1) [33]. MXenes with surface functional groups such as F, OH, and O are capable of strong interaction with other semiconductors to generate efficient heterojunctions. MXenes (Ti_3_C_2_) successfully adsorb the hazardous pollutants and organic dyes, providing effective adsorbents for the removal of dyes from wastewaters [28,34]; their layered and porous structures facilitate their adsorption and storage capabilities [19,35]. Several hybrid structures have been constructed from MXenes for photocatalytic purposes [36,37]. For instance, rice crust-like hybrid structures were constructed from zinc oxide (ZnO) and MXene (Ti_3_C_2_T_x_) with excellent photocatalytic performance, showing large surface area, Schottky barrier generation, and ideal band alignment. Besides, these hybrid photocatalysts showed excellent recyclability and stability [36]. Herein, the most recent developments pertaining to the photocatalytic degradation of organic and pharmaceutical pollutants are deliberated, with a focus on important challenges and future perspectives; important techniques for synthesis of these photocatalysts are also covered.

## 2. Techniques for the Synthesis of MXene-Based Photocatalysts

Several techniques have been introduced for synthesizing a variety of MXene-based (nano)photocatalysts, including electrostatic self-assembly, calcination processes, hydrothermal treatment, mechanical/ultrasonic mixing, solvothermal treatment, etc. [49,50]. Among them, mechanical/ultrasonic mixing techniques have the advantage of simplicity when preparing MXenes; robust mechanical stirring and/or high-power ultrasonic vibration has been typically applied for retaining the intimate contact between MXenes and photocatalysts [49]. In one study, hydrogels of Ti_3_C_2_T_x_ were prepared through the stirring of MXenes and graphene oxide colloidal solutions with Eosin Y solution for 0.5 h; after that the mixtures were kept at 70 °C with the protection of N_2_ gas [51]. In another study, cadmium sulfide (CdS)/MXene (Ti_3_C_2_) composites were synthesized using an electrostatic self-assembly technique. Accordingly, MXene nanosheets and CdS nanowires were well-combined using electrostatic attraction, and the nanowires were successfully distributed on MXene nanosheets [52]. Notably, hydrothermal and solvothermal methods have been broadly applied for producing MXene-based composites. For instance, an in situ metal organic framework (MOF)-derived technique was deployed for synthesizing Co-Co layered double hydroxide/Ti_3_C_2_T_X_ nanosheets with photocatalytic potentials using a solvothermal method [53]. Besides this, Ti_3_C_2_/Bi_2_WO_6_ composites were synthesized using electrostatic attraction and a hydrothermal method; these composites exhibited efficient photocatalytic performance with enhanced CO_2_ adsorption potential [54]. 

In etching techniques, a variety of etchants can be applied for fabricating MXenes, including hydrogen fluoride (HF), lithium fluoride (LiF) plus HF, zinc chloride (ZnCl_2_), etc. Different MXenes could be obtained by adjusting the concentration and duration of the etching process [55,56]. For instance, the replacement reaction method was deployed for synthesizing Zn-based MAX and Cl-terminated MXenes using ZnCl_2_ Lewis acidic molten salt. However, several challenging issues ought to be considered regarding higher temperature, poor crystallinity, purity requirements, and high energy consumption [57]. In addition, several chemical vapor deposition techniques have been reported for controlled manufacturing of MXene epitaxial films with multilayers [58]. In one study, 2D ultrathin α-Mo_2_C crystals with a large surface area were synthesized through a chemical vapor deposition technique, exhibiting high quality with defect-free structures [59]. Besides this, 2D Mo_2_C was directly synthesized on grown graphene (in situ) deploying a one-step copper-catalyzed chemical vapor deposition technique [60]. Despite the advantages of chemical vapor deposition techniques such as the fabrication of MXenes with low impurity and defects, low yield of production and complex treatment pathways are some of their crucial drawbacks; with additional modifications, these techniques can be further improved [61]. 

## 3. Photocatalytic Degradation of Organic and Pharmaceutical Pollutants

Photocatalysis is considered a relatively safer and low-cost strategy for the elimination of hazardous pollutants [37,62,63]. In this context, MXenes with distinctive lamellar structures and remarkable conductivity can be applied as nano-adsorbents to remediate environmentally toxic pollutants, and also as co-catalysts for improving photocatalytic degradation potential of the other composites in their photocatalytic performances [13,64]; several studies have focused on photocatalytic degradation of dyes, heavy metals, and pharmaceutical pollutants. For instance, MXene/TiO_2_ nanocomposites were constructed for photocatalytic degradation of organic pollutants [65]. In one study, MXene (Ti_3_C_2_) nanosheets decorated with copper sulfide (CuS) particles were fabricated using a hydrothermal treatment technique to enhance the catalytic persulfate activation under light, showing excellent reactivity for rapid elimination of Orange II; the CuS@MXene composites exhibited high stability even after 4 cycles of reusability evaluation [66]. The dosage of persulfate and initial pH of solution were the chief parameters affecting the elimination rate of pollutant; ^1^O_2_ was the primary reactive species for removing Orange II using the nanocomposite [66]. 

**Figure 2:**
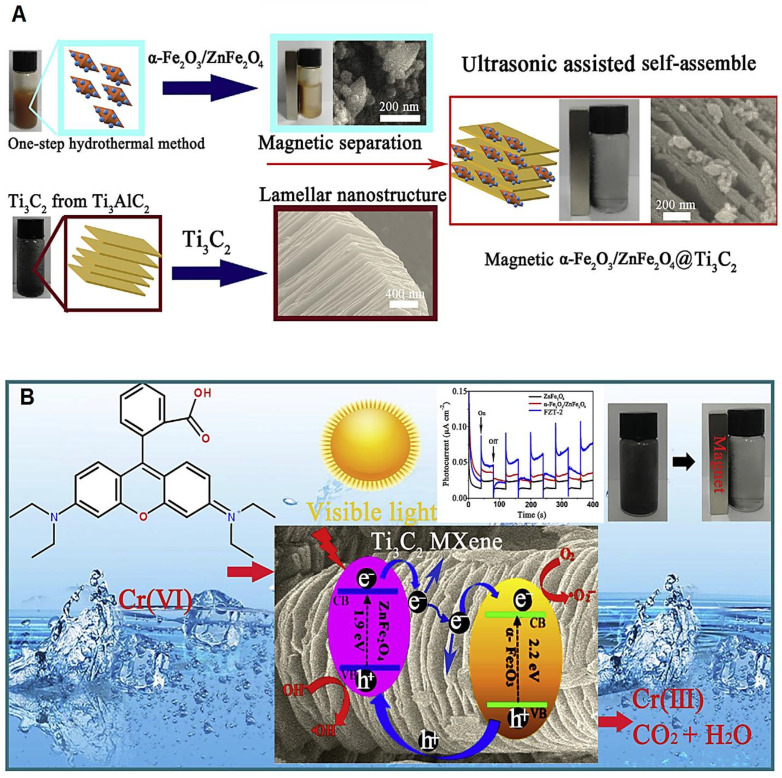
(**A**) The preparative process of magnetic α-Fe_2_O_3_/ZnFe_2_O_4_@MXene (Ti_3_C_2_) composites, with (**B**) mechanism of rhodamine B and toxic Cr (VI) photocatalytic removal in water. Adapted from Ref. [67] with permission. Copyright 2019 Elsevier.

After the formation of magnetic heterojunctions of α-Fe_2_O_3_/ZnFe_2_O_4_ through a simple hydrothermal fabrication technique, the photocatalyst was prepared utilizing MXenes as co-catalysts through ultrasonic-assisted self-assembly to disperse obtained magnetic heterojunctions on the surface of MXene (Ti_3_C_2_) (Figure 2) [67]. These photocatalysts exhibited improved photocatalytic activity in elimination of rhodamine B and toxic Cr (VI) in water with reusability and high conductivity advantages [67]. In another study, MXene (Ti_3_C_2_)/MoS_2_ nanocomposites with a specific surface area were prepared via a hydrothermal approach, showing efficient photocatalytic organic pollutant degradation [68]. These MXene-based nanophotocatalysts with excellent removal of methyl orange pollutants exhibited improved optical absorption potential. Notably, the incorporation of MXenes with MoS_2_ nanosheets could improve the photocurrent response and reduce the electrochemical impedance, causing an improvement in electron transfer of excited semiconductors along with an inhibition in charge recombination [68]. 

**Figure 3:**
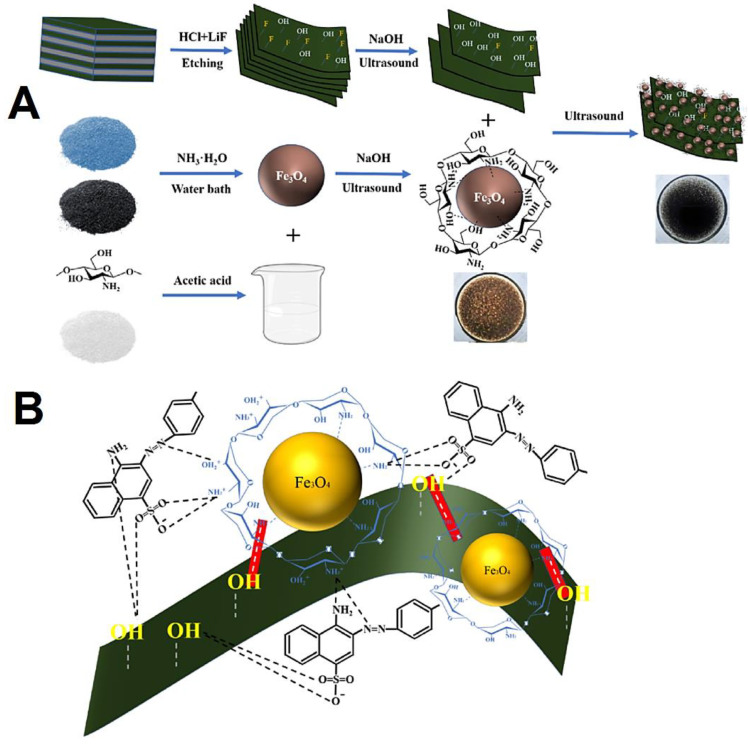
(**A**) The preparative process for MXene@Fe_3_O_4_@chitosan (CS) magnetic nanocomposites, with related adsorption mechanism (**B**). LiF: lithium fluoride. Adapted from Ref. [74] with permission. Copyright 2022 Elsevier.

The hybridization strategies can help to enhance the photocatalytic degradation efficiency of MXenes and their derivatives [16]. Several studies have focused on hybridization of MXenes with other materials, including graphene and its derivatives, metal–organic frameworks (MOFs), polymers (cellulose or chitosan), carbon nanotubes, carbon dots, etc. [69,70,71]. MXenes hybridized with graphene and their derivatives have been studied due to their fascinating properties. In one study, graphene oxide-based nanofiltration membranes intercalated with TiO_2_ nanomaterials were prepared by the in situ oxidation of MXene nanosheets; ensued composites could remove the organic dyes with high efficiency (~97%) [72]. In addition, graphitic carbon nitride (*g*-C_3_N_4_) and TiO_2_ were attached to the graphene layers, and then were applied for synthesizing photocatalysts using MXenes (Ti_3_C_2_) via a simple calcination process (in situ); these photocatalysts had excellent degradation potential for pharmaceutical and organic pollutants under visible light irradiation, including tetracycline, ciprofloxacin, rhodamine B, and bisphenol A [73]. Notably, these stable and reusable photocatalysts had improved visible light adsorption and photo-generated carrier separation. The visible light photocatalytic degradation rate was significantly improved, because of the synergist effects and robust interactions among *g*-C_3_N_4_, graphene, and TiO_2_ for the separation and accumulation of *g*-C_3_N_4_ electrons with superb reduction potential and TiO_2_ holes with remarkable oxidation ability, thus making them as promising candidates for pharmaceutical degradation/elimination [73]. Xu et al. [74] constructed MXene@Fe_3_O_4_@chitosan composites using an ultrasonic self-assembly technique for removing Congo red (Figure 3); chitosan with adsorption capacity of 620.22 mg·g^−1^ was deployed for improving the adsorption capacity. Accordingly, the adsorption surveyed Langmuir isotherm model, and the related kinetics were detected to mainly follow the quasi-secondary rate kinetics. Notably, it was revealed that adsorption was endothermic, entropy-driven, and based on the thermos-dynamically spontaneous process. The magnetic nanocomposites exhibited significant adsorption capacity under neutral or weakly acidic conditions; however, under alkaline conditions, they displayed very low adsorption capacity [74]. 

**Figure 4:**
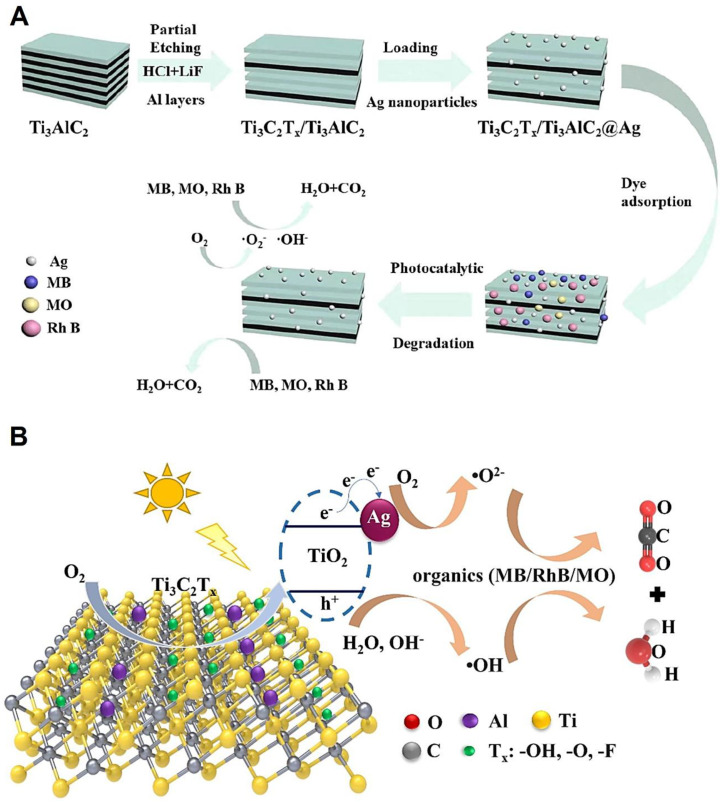
(**A**) The preparative process of ternary Ti_3_C_2_T_x_/Ti_3_AlC_2_@Ag composites for the photocatalytic degradation of pollutants. (**B**) ROS and photodegradation reaction mechanisms. MB: methylene blue; RhB: Rhodamine B; MO: methylene orange. Adapted from Ref. [76] with permission. Copyright 2022 Elsevier.

Structures constructed from MXene (Ti_3_C_2_)/O-doped *g*-C_3_N_4_ Schottky-junction were reported through an in situ electrostatic assembly of negatively charged MXenes and positively charged O-doped *g*-C_3_N_4_ nanosheets. The obtained photocatalysts exhibited enhanced hydrogen evolution and photocatalytic activity due to the synergy between the compounds as well as the formation of a Schottky-junction [75]. In one study, ternary Ti_3_C_2_T_x_/Ti_3_AlC_2_@Ag composite photocatalysts were fabricated for catalytic degradation of methylene blue, Rhodamine B, and methylene orange, with an efficiency of 99.7%, 98.9% and 99.3%, respectively (Figure 4) [76]. Synergistic effects could be obtained between Ag nanomaterials and partial etched Ti_3_C_2_Tx/Ti_3_AlC_2_ nanosheets, which were illustrated by transference of the photogenerated carriers and the formation of reactive oxygen species (ROS); these effects could additionally enhance the catalytic degradation performance [76].

MXene-based nanocomposites fabricated via catalytic chemical vapor deposition were hybridized with carbon nanotubes for photocatalytic removal of Rhodamine B, wherein the photocatalyst degradation efficiency for pure MXenes and MXene-carbon nanotube composites were found to be 60% and 75%, respectively. The hybrid composites exhibited good degradation efficiency for pollutants in successive cycles [77]. In addition, MXene-based composites comprising multi-layered MXene (Ti_3_C_2_) nanosheets and transition metal oxide nanomaterials, i.e., zinc oxide, bismuth molybdate and tin dioxide, were introduced for the catalytic removal of pollutants with high adsorption capacity towards methylene blue [78]. Accordingly, 150 mg L^−1^ of high concentration methylene blue solution and 20 mg L^−1^ of 4-chlorophenol solution were totally eliminated by adsorption and photocatalysis using these MXene-based photocatalysts for 4 h under visible light irradiation. Studies on mechanisms revealed that hydroxyl and superoxide free radicals were reactive species in the degradation procedure; coupling transition metal oxide and MXene inhibited the recombination of photogenerated electron–hole pairs [78]. These MXene-based nanocomposites with synergistic removal of pollutants and strong adsorption thus offer themselves as promising photocatalysts for environmental pollution control.

The design of heterostructures can improve the catalytic performance of MXene-based photocatalysts, because of the triggered interfacial electron transfer [64]. In one study, the chimeric ZnO nanosheets with suitable carrier mobility were created on the accordion-shaped surface of MXenes (Nb_2_C and V_2_C) to obtain hierarchical ZnO/MXene heterostructures as photocatalysts for degradation of dyes [79]. These structures with good stability exhibited improved photocatalytic activity for degrading methylene blue; the degradation rate for ZnO/Nb_2_C was ~62.62% and for ZnO/V_2_C was found to be ~99.53, after UV irradiation within 120 min. The degradation rate endured at 58.6% and 97.3%, respectively, after 4 cycles, while it was only 22.0% for pure ZnO. This is because of the formed ZnO/MXene hierarchical structures with uniform active sites on their surfaces and porous structures, which prevented the aggregation of ZnO or MXenes. Notably, the heterostructures between ZnO and MXenes could abridge the carrier transfer pathway, facilitating the transfer of photo-generated electrons from the conduction band of ZnO to the conduction band of MXenes, and enhancing the efficiency of photocatalytic degradation [79]. This strategy can improve the catalytic activity of other 2D photocatalytic structures, paving a way for designing efficient photocatalysts. Sun et al. [80] developed MXene (Ti_3_C_2_)-bridged Ag/Ag_3_PO_4_ hybrid composites with Ti_3_C_2_ content-dependent photocatalytic performance for the degradation of MO and Cr(VI) reduction under visible light irradiation; the highest MO degradation efficiency could be obtained with 3 wt% Ti_3_C_2_ content after irradiation for 1 h. The mechanistic studies revealed that improved photocatalytic performance was associated with the synergetic effects that originated from Ag, Ag_3_PO_4_, and MXenes, enhancing visible light absorption and promoting separation and transfer of photo-generated electron–hole pairs [80]. 

**Figure 5:**
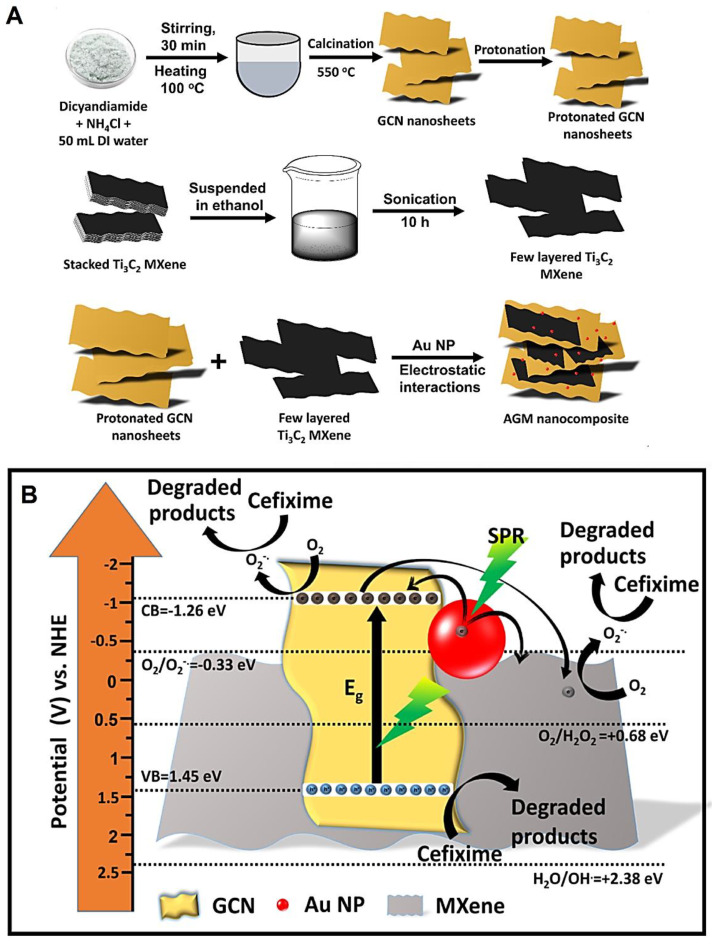
(**A**) The preparative process of Au nanomaterial-decorated *g*-C_3_N_4_/MXene (AGM) nanocomposites. (**B**) The photocatalytic decomposition mechanism for pharmaceutical pollutant (cefixime) using the designed nanocomposites under visible light illumination. GCN: *g*-C_3_N_4_; SPR: surface plasmon resonance. Adapted from Ref. [82] with permission. Copyright 2022 Elsevier.

Photocatalytic removal of pollutants of pharmaceutical origin and industrial wastes has been explored using MXene-based composites. For instance, MXene-Ti_3_C_2_/MoS_2_ composites fabricated through a hydrothermal treatment technique were deployed for the photocatalytic removal of ranitidine and reduction of nitrosamine dimethylamine generation potential under visible light irradiation [81]. A heterojunction could be detected between MoS_2_ and MXene, facilitating the separation of electron–hole pairs and charge transfer to improve photocatalytic activity. These composites demonstrated superior photocatalytic activity within ~60 min; the highest ranitidine degradation (~88.4%) and mineralization (~73.58%) efficiency, and the lowest nitrosamine dimethylamine formation potential of only ~2.01%. Mechanism studies revealed that active species such as •O_2_^−^ radicals, h^+^ and •OH radicals were responsible for degrading ranitidine, and •OH radicals were the chief active species that were responsible for photocatalytic performance [81]. Kumar et al. [82] developed MXene (Ti_3_C_2_) coupled *g*-C_3_N_4_ nanosheets based on plasmonic photocatalysts with good reusability (up to 3 cycles) for removal of pharmaceutical pollutants (cefixime) under visible light irradiation (Figure 5). After the optimization process, the photocatalysts containing 3 wt% MXenes could efficiently remove (~64.69%) cefixime under visible light exposure within 105 min. Mechanistic studies revealed that the presence of gold (Au) nanomaterials and MXenes in these nanocomposites could facilitate the outstanding charge carrier separation and increase the number of active sites, because of the generation of interfacial contact with *g*-C_3_N_4_ nanosheets. In addition, the plasmonic influence of Au nanomaterials improved the absorption of light photons, which could enhance the photocatalytic activity of these nanocomposites. Nanosheets of *g*-C_3_N_4_ were employed as semiconducting photocatalysts to efficiently harvest energy from visible light. Besides this, MXene nanosheets functioned as superb electron sinks and provided improved surface areas for the adsorption of hazardous pollutants; the application of MXenes as co-catalysts in these nanocomposites facilitated the interfacial separation of charge carriers due to their excellent metallic conductivity and their abundant functional groups [82].

**Figure 6:**
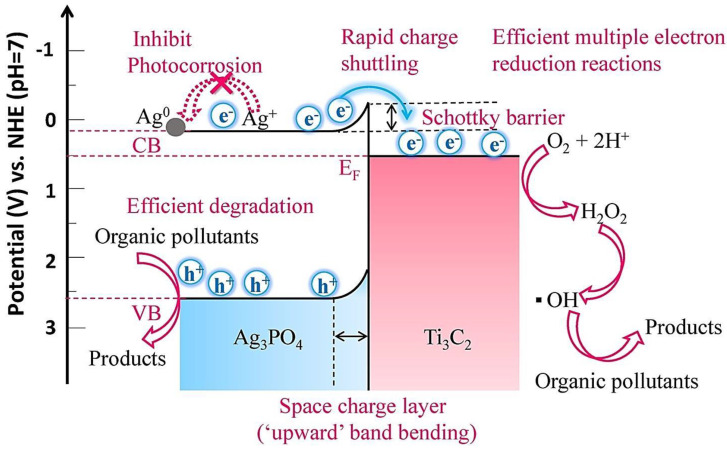
The possible mechanism of photocatalytic degradation of organic pollutants along with photo-corrosion inhibition of Ag_3_PO_4_/MXene (Ti_3_C_2_) Schottky catalysts. Adapted from Ref. [88] with permission. Copyright 2018 Elsevier.

The Z-scheme heterojunction-based photocatalyst has advantages of excellent electron–hole pairs separation efficiency, robust redox potential, and a broad light response range; the significant power of oxidization and reduction makes the Z-scheme heterojunction suitable for degradation of pollutants in water [83,84,85]. In one study, a Z-scheme heterojunction of *g*-C_3_N_4_/MXene (Ti_3_C_2_)/MoSe_2_ was developed to achieve effective visible light-induced removal of enoxacin within 60 min [86]. Accordingly, MXene-stimulated interface electron separation could play an important role on degradation with high efficiency. Furthermore, MXene as a suitable mediator between *g*-C_3_N_4_ and MoSe_2_ could effectively transfer the electron and inhibit its recombination. Notably, a Schottky-junction could be generated between MXene (the conductor) and *g*-C_3_N_4_/MoSe_2_ (the semiconductor) to improve electron trapping; the trapped electron could stimulate the active species generation to enhance performance. During degradation, most commonly produced reactive species were **•**O_2_^−^ and photogenerated electrons. The prepared photocatalyst exhibited promising degradation potential for other pollutants, including norfloxacin (~80%), moxifloxacin (~100%), ofloxacin (~100%), gatifloxacin (~65%), levofloxacin (~100%), and ciprofloxacin (~80%) [86]. Cao et al. [87] synthesized *g*-C_3_N_4_/TiO_2_@Ti_3_C_2_ photocatalysts for effective visible light degradation of pollutants such as MO, revealing higher degradation rates with improved photocatalytic performance owing to the Z-type heterojunctions in the nanocomposites. It is because of the close interaction among the multilayer MXenes, perforated *g*-C_3_N_4_ flake with a large specific surface area, and oxidation-generated TiO_2_ promoting the separation of photocatalytic carriers and catalytic reactions [87]. MXenes can highly improve the catalytic performance and stability of materials such as Ag_3_PO_4_ due to the abundant surface hydrophilic functional groups forming robust interfacial contact with Ag_3_PO_4_, facilitating the separation of carriers [88]. Additional radical **•**OH formation is also stimulated by the strong redox reactivity of surface Ti sites promoting multiple electron reduction reactions. In one study, a Schottky-junction generated at Ag_3_PO_4_-MXene (Ti_3_C_2_) interface suitably transferred electrons to the surface of the MXene by a built-in electrical field (Figure 6). The prepared composites displayed high photocatalytic degradation capabilities towards organic pollutants such as 2,4-dinitrophenol. The photocatalytic degradation of tetracycline hydrochloride was also maintained at ~68.4%, even after 8 successive cycles of reuse [88]. 

MXenes and MOFs have been hybridized for the photocatalytic degradation of pollutants. Long et al. [89] designed MXene (Ti_3_C_2_)/NH_2_-MIL-88B(Fe) composites with a photocatalytic removal potential (>90%) towards methylene blue (50 ppm) within 30 min, along with the elimination of ~83.41% Rhodamine B (25 ppm) and 44.26% MO (20 ppm) within 120 min. The use of MXenes could help to prevent the agglomeration of MOFs and enhance the effective area of the composite material, acting as a photo-generated electron trap in the photocatalytic procedure for improving the charge separation efficiency of MOFs. The main mechanisms for dye removal entailed adsorption performance and the Schottky knot synergistic Fenton effect [89]. Since MXenes (Ti_3_C_2_) typically cannot be directly applied for a photocatalysis process and this matter may restrict their additional photocatalytic applications (especially in water), the proposed hybridization strategy using MOFs can help to improve their properties and functionalities. Another hybrid MXene-based photocatalyst was introduced using NiCo_2_S_4_ and MXene [90]. The photocatalyst with great photostability in 5 repetitive runs exhibited efficient degradation of rhodamine B (~100%) under visible light irradiation within 20 min. It was revealed that rhodamine B was primarily oxidized into non-toxic products such as H_2_O, CO_2_, and a few inorganic species, offering new opportunities for wastewater management with advantages of environmentally benign properties, cost-effectiveness, and the formation of non-toxic by-products [90]. 

## 4. Conclusions and Perspectives

MXene-based composites have attracted much attention as photocatalysts to degrade pollutants owing to their unique optical/thermal features, hydrophilicity, large surface area, surface controllable chemical properties, high chemical stability, regular planar architectures, high metal conductivity, and abundant derivatives. However, rapid recombination of photo-generated carriers typically may lead to a reduction in their photocatalytic functions. Designing a heterostructure can effectively improve the photocatalytic activities of MXene-based composites, because of the stimulated interfacial electron transfer. Notably, hybridization of MXenes with other materials such as MOFs, graphene, and polymers can help to improve their photocatalytic properties. In addition, MXenes with unique lamellar structures and excellent conductivity have been deployed as co-catalysts for enhancing the photocatalytic degradation capabilities of composites. Several aspects need to be systematically studied, including finding the functional groups’ orientation on their surfaces. With advancements in photocatalysis and nanotechnology, MXenes and their derivatives with unique structural and physicochemical properties along with their biocompatibility, robust electrochemistry, and hydrophilicity can be employed as multifunctional candidates in novel environmental clean-up technologies for detection, mitigation, and elimination of various hazardous contaminants and organic/inorganic pollutants from environmental matrices; finding suitable techniques for improving the properties and functionality of MXene-based (nano)photocatalysts is one of the most important challenges ahead. More analytical and feasibility studies are still needed to move from laboratory-stage experiments to large/industrial-scale remediation of pollutants using MXenes. In addition, since Ti_3_C_2_T_x_ is the most studied MXene for photocatalytic applications, it is suggested that future studies move towards the design of novel photocatalysts using other types of MXenes. The stability of MXenes still needs additional adjustments using suitable functionalization and/or post-production optimization processes. Large-scale production of MXene-based photocatalysts is another crucial challenging issue; finding optimal reaction/synthesis conditions (pH, temperature, pressure, etc.) with advantages of simplicity, cost-effectiveness, eco-friendly properties, and repeatability can help move towards large-scale and commercial production of these photocatalysts. 

## Figures and Tables

**Table 1:** Some selected MXene-based composites with photocatalytic degradation performance towards organic and pharmaceutical pollutants.

MXene-Based Photocatalysts	Pollutants	Advantages/Properties	Refs.
Carbon nitride coupled with Ti_3_C_2_-Mxene derived amorphous titanium (Ti)-peroxo heterojunction	Rhodamine B and tetracycline	- High reusability and stability - Superb photocatalytic degradation efficiency over Rhodamine B (~97.22%) and tetracycline (~86.34%) under visible light irradiation (λ > 420 nm), within 60 min	[38]
Carbon/Ti-based oxide nanocomposites/MXenes (Ti_3_C_2_T_x_)	Methylene blue	- Significant removal of methylene blue (~90%) under visible light illumination, within 1 h. - Narrowed band-gap with suitable electronic conductivity - Large specific surface area - Excellent efficiency of charge separation with improved absorption capabilities	[39]
Rod-like Nb_2_O_5_/Nb_2_CT_x_ composites	Rhodamine B and tetracycline	- High photocatalytic degradation of Rhodamine B (~98.5%, 120 min) and tetracycline (~91.2%, 180 min) under visible light irradiation - High photoactivity and cycle stability	[40]
TiO_2_/Ti_3_C_2_T_x_	Carbamazepine	- High photocatalytic degradation of carbamazepine (~98.67%) under UV light irradiation - •OH and •O_2_ attacked the carbamazepine molecule during the photocatalytic degradation	[41]
Lanthanum (La)- and manganese (Mn)-co-doped bismuth ferrite/MXene (Ti_3_C_2_) composites	Congo red	- Fast degradation of organic dye molecules - ~92–93% degradation of dye pollutant	[42]
MXene (Ti_3_C_2_)/Ag_2_ZnGeO_4_ Schottky heterojunctions	Acid red 1	- Enhanced visible light photocatalytic performance - Improved formation of superoxide radicals as the chief active species - Efficient charge separation	[43]
MXene-based catalysts (TiO_2_/Ti_3_C_2_) modified with silver (Ag) particles	Nitrate	- The photoelectron density as well as the carrier separation of the catalysts could be improved by applying Ag particles - The rate of nitrate elimination ~96.1%; the selectivity towards nitrogen was ~92.6% - High photocatalytic removal efficiency (~89%) towards nitrate, even after 5 cycles	[44]
Magnetic-Ti_3_C_2_T_X_	Diclofenac	- Enhanced degradation of diclofenac due to the formation of active radicals, including the hydroxyl radical and reactive chlorine species - Excellent stability and photodegradation efficiency during seven consecutive degradation reaction cycles	[45]
Ti_3_C_2_/*g*-C_3_N_4_	Phenol	- 98% phenol removal efficiency - 32.1% phenol could be degraded under dark conditions owing to the capability of MXenes in storing additional photo-generated electrons under light irradiation and releasing them when exposed to electron acceptors in dark conditions	[46]
CuFe_2_O_4_/Ti_3_C_2_	Sulfamethazine	- The synergistic degradation effects under visible light - The breaking of S–N bonds could be detected; the oxidation of aniline and deamination were dominated by attacking •OH	[47]
Bismuth/bismuth oxychloride (Bi/BiOCl) microspheres/Ti_3_C_2_	Ciprofloxacin	- Excellent reusability in the 5 cycles of ciprofloxacin degradation - High photocatalytic performance	[48]

## Data Availability

Not applicable.
